# Taiwan cobra envenoming: serum venom concentration before and after specific treatment and relationship with debridement of necrotic wound tissue

**DOI:** 10.1590/1678-9199-JVATITD-2022-0027

**Published:** 2023-01-13

**Authors:** Chia-Cheng Wang, Chun-Hsiang Ou Yang, Chih-Po Hsu, Chien-Chun Liu, Jau-Song Yu, Chih-Hong Lo, Wen-Chih Fann, Yen-Chia Chen, Chih Chuan Lin

**Affiliations:** 1 Department of Traumatology and Emergency Surgery, Chang Gung Memorial Hospital, Chang Gung University, Taoyuan, Taiwan.; 2 College of Medicine, Chang Gung University, Taoyuan, Taiwan.; 3 Molecular Medicine Research Center, Chang Gung University, Taoyuan, Taiwan.; 4 Department of Cell and Molecular Biology, College of Medicine, Chang Gung University, Taoyuan, Taiwan.; 5 Liver Research Center, Chang Gung Memorial Hospital, Linkou, Taiwan.; 6 Research Center for Food and Cosmetic Safety, Research Center for Chinese Herbal Medicine, College of Human Ecology, Chang Gung University of Science and Technology, Taoyuan, Taiwan.; 7 Department of General Surgery, Chang Gung Memorial Hospital, Taoyuan, Taiwan.; 8 Department of Emergency Medicine, Chiayi Chang Gung Memorial Hospital, Chiayi, Taiwan.; 9 Department of Emergency Medicine, Taipei Veterans General Hospital, Taipei, Taiwan.; 10 Department of Emergency Medicine, School of Medicine, National Yang-Ming University, Taipei, Taiwan.; 11 Department of Emergency Medicine, Linkou Chang Gung Memorial Hospital, Taoyuan, Taiwan.

**Keywords:** Taiwan cobra, Naja atra, Venom concentration, Wound necrosis, Wound debridement

## Abstract

**Background:**

Bivalent freeze-dried neurotoxic (FN) antivenom has been the primary treatment since the 1980s for Taiwan cobra (*Naja atra*) envenomation in Taiwan. However, envenomation-related wound necrosis is a significant problem after cobra snakebites. In the present study, we analyzed the changes in serum venom concentration before and after antivenom administration to discover their clinical implications and the surgical treatment options for wound necrosis.

**Methods:**

The patients were divided into limb swelling and wound necrosis groups. The clinical outcome was that swelling started to subside 12 hours after antivenom treatment in the first group. Serum venom concentrations before and after using antivenoms were measured to assess the antivenom's ability to neutralize the circulating cobra venom. The venom levels in wound wet dressing gauzes, blister fluids, and debrided tissues were also investigated to determine their clinical significance. We also observed the evolutional changes of wound necrosis and chose a better wound debridement timing.

**Results:**

We prospectively enrolled 15 Taiwan cobra snakebite patients. Males accounted for most of this study population (n = 11, 73%). The wound necrosis group received more antivenom doses than the limb swelling group (4; IQR:2-6 vs 1; IQR:1-2, p = 0.05), and less records of serum venom concentrations changed before/after antivenom use (p = 0.0079). The necrotic wound site may release venom into circulation and cause more severe envenomation symptoms. Antivenom can efficiently diminish limb swelling in cobra bite patients. However, antivenom cannot reduce wound necrosis. Patients with early debridement of wound necrosis had a better limb outcome, while late or without debridement may have long-term hospital stay and distal limb morbidity.

**Conclusions:**

Antivenom can efficiently eliminate the circulating cobra venom in limb swelling patients without wound necrosis. Early debridement of the bite site wound and wet dressing management are suggestions for preventing extended tissue necrosis and hospital stay.

## Background

Cobra bite is a critical issue when considering snakebite as a neglected tropical disease [1]. In Taiwan, Taiwan cobra or *Naja atra* envenomation accounts for approximately 20% of snakebite cases, but almost no mortality [2, 3]. In the 1980s, the Vaccine Center of the Center for Disease Control of Taiwan developed the bivalent freeze-dried neurotoxic (FN) antivenom used to treat Taiwan cobra bitten patients [4, 5]. However, the treatment guidelines provided by the Taiwan National Poison Control Center (TPCC) for Taiwan cobra bite patients were based mainly on expert opinions and animal study results [6]. The guideline stated only a rough range of dosage of FN antivenom (6-10 vials) for envenomed patients and no mention of any recommendation in treating Taiwan cobra bite-related wound necrosis. Unfortunately, up to our knowledge, there were only observational or retrospective studies regarding treating Taiwan cobra bite patients in the medical literature [7, 8, 9]. With few neurotoxic symptoms, Taiwan cobra envenomation primarily causes local tissue swelling and tissue necrosis in some cases [7, 8]. Physicians use the FN antivenom to prevent the progression or reduce limb swelling in Taiwan cobra envenomed patients.

Nevertheless, the snakebite necrotic wound requiring surgical procedures have remained high even with large doses of FN antivenom administrated in cobra bite patients [7, 8, 9, 10]. In the present study, we observed and analyzed the changes in serum venom concentrations before and after antivenom administration to discover their clinical implications in Taiwan cobra envenomated patients. The venom levels in wound wet dressing gauzes, wound blister fluids, and debrided tissues were also investigated to determine their clinical significance. The results would help develop rational countermeasures in treating Taiwan cobra snakebite patients.

## Methods

### Study design and inclusion criteria

This is a prospective observational study. The patients enrolled in this research aged over 18 years, were bitten by Taiwan cobra snakes, and presented themselves to the emergency departments (ED) of Linkou Chang Gung Memorial Hospital, Chiayi Chang Gung Memorial Hospital, and Taipei Veterans General Hospital between January 2019 to January 2021. If patients came from other hospitals with initial antivenom treatment, they might also be included in this study. If the patient refused to join the study or refused further treatment in ED or after admission, they were excluded from the research. All participants of the study signed an informed consent form.

### Ethics statement

This study was approved by the Institutional Review Board (IRB) of Taipei Veteran General Hospital (approval no.: 2019-12-003A) and Linkou Chang Gung Memorial Hospital (IRB no. 201801542B0A3).

### Collection of demographic, laboratory, and clinical data

The collected variables were as follows. Demographic variables were age, gender, and snakebite information, including the culprit snake, biting time, and patient's presentation time (minutes, defined as the time since they got bitten during ED visits). Patients were asked to identify the culprit snake through a pictorial atlas of the six medically essential snakes in ED. The species of the culprit offending snake were also determined by the ELISA method we developed earlier [11]. Clinical symptoms and signs such as the location and degree of limb swelling, symptom progression, and necrosis were recorded. Photos of the patient's wound were taken when they were admitted to ED or at any following times after the administration of antivenom and when the affected limbs reached maximum swelling.

### Classification of snakebite severity in patients bitten by Taiwan cobra

Since there is no consensus on the severity grading for snakebite envenomation in Taiwan, we defined a limb swelling scale based on modifying a previously published severity score [12]. The scale system has three parameters:

1. Swelling and erythema.
2. The number of joints crossed.
3. Wound necrotic change.

We defined four degrees of severity in snakebite patients (Table 1):

1. Dry bite (degree 0): the dry bite was defined as no swelling or erythema around the fang marks/biting site.
2. Mild degree (degree 1): The mild degree was defined as limb swelling or erythema limited to the surrounding area and was equal to or less than 10 cm in size without one joint involvement.
3. Moderate degree (degree 2): The moderate degree was defined as swelling and erythema around the fang marks were 10-20 cm and one joint involvement (wrist or ankle) and/or small necrotic change (less than 2 cm in diameter).
4. Severe degree (degree 3) was defined as limb swelling and erythema greater than 20 cm, extended over two joints, and/or significant local tissue necrosis.

**Table 1.** Classification of severity degree of snakebite patients in the emergency departments of Taiwanese hospitals.

Severity degree	Parameter			
	Swelling and erythema	Number of joints crossed	Wound necrotic change	Suggested dose of antivenom
0 - Dry bite	None	None	None	No need
1 - Mild degree	< 10 cm	None	None	1 vial
2 - Moderate	10-20 cm	1	< 2 cm necrosis	2 vials
3 - Severe	> 20 cm	2	> 2 cm necrosis	3 vials or more

### Suggested dose of antivenom

Antivenom administration followed the indications of antivenom of World Health Organization guidelines [13]. The ED physicians determined the dose of the administrated antivenom according to the degree of severity as none (dry bite), one (mild degree), two (moderate degree), and three vials (severe degree). After administrating antivenom, patients were monitored in ED to see if there was a clinical improvement in limb swelling or systemic neurotoxic effects. No more antivenom will be added if the limb swelling is stopped or no progression more than another joint within 6 hours after the first dose of antivenom used in our ED or other hospitals (beyond elbow and knee, respectively, if a patient is bitten in hand/foot/wrist and ankle). On the contrary, those whose limb swelling progresses beyond another joint within six hours can be treated with another vial of antivenom. However, if the swelling goes rapidly, then another or more vials of antivenom could be administrated depending on the clinical judgment of ED physicians.

### Decision-making process of surgical incision, debridement or fasciotomy

 Surgical interventions such as debridement, fasciotomy, etc., were performed with clinical assessment. In the past, surgical procedures were usually performed three days later, except compartment syndrome occurred. However, we found that tissue necrosis may extend after the time in this clinical observation. So, the early surgical procedure was performed in the least cases for clinical benefit. The procedure may also differ in different sites of wound necrosis because soft tissue loss, tendon, or vassal exposure may need to be considered for further wound care. If the necrosis is in the distal part of a limb (finger, toe, hand, and foot), limited incision and debridement may be performed; if the necrosis site is in a proximal limb or much soft tissue, wide excision may be performed to avoid the necrosis extension.

### ELISA-based venom detection method and detection of serum venom concentration

The venom concentration of the victim's serum was measured by the venom-detected ELISA assay described previously **[** 10 **]**. Briefly, a serum sample (100 μL) was added into the microplate coated with capture antibodies recognizing neurotoxic (*B. multicinctus* and *N. atra*) venom-specific proteins and incubated for 30 min. at room temperature (RT). After washing six times with phosphate-buffered saline with Tween 20 (PBST), 100 μL of detection antibodies with Horseradish peroxidase (HRP) labeling, diluted 1:16000 in PBS, was added onto the microplate and incubated for 30 mins. This microplate was washed six times with PBST again. Then, 3,3'5,5'-tetramethylbenzidine (TMB) buffer was added to each well and incubated for 10 mins, stopping the reaction by 2N H_2_SO_4_. A SpectraMax M5 microplate reader measured the absorbance of each well at a wavelength of 450 nm. The venom concentration of each sample was calculated according to the standard curves of crude hemorrhagic venoms. The limits of quantification (LOQ) of the ELISA for neurotoxic venoms were determined to be 0.39 ng/mL.

Blood samples were drawn to determine the serum venom concentration to determine the effect of antivenom on neutralizing the circulating venom before antivenom and 6 hours after the first vial of antivenom (pre-antivenom and post-antivenom concentrations). If a patient was referred from other hospitals, no blood sample was collected for the pre-antivenom venom concentration if they received FN antivenom in the first-aid hospital. However, the serum post-antivenom concentrations was determined. 

The venom levels in wound wet dressing gauzes, wound blister fluids, and debrided tissues were also investigated to determine their clinical significance. The gauze (5 cm × 5 cm) was soaked into 1 mL PBS within 5 mL Eppendorf and sonicated for 10 min. in RT. Then, the buffer in the gauze was squeezed out and collected in 1.5 mL Eppendorf. This body fluid-containing buffer was stored at -80 degrees before use. The frozen tissue (about 1 cm^3^) was homogenized in 250 μL of PBS using Precellys 24 Homogenizer (Bertin Instruments, France) according to the manufacturer's instructions. The supernatant was stored at -80 degrees before use.

### Outcomes

The clinical outcome was limb swelling began to be released 12 hours after antivenom treatment for patients with limb swellings but without wound necrosis. We observed the degree of limb swelling and conditions of tissue necrosis since the patient came to our ER. If needed, the surgical procedure may be performed when tissue necrosis area progresses. All patients were followed up at least once in the outpatient clinic one week after ED or ward discharge. Patients' clinical conditions determined more times of outpatient clinic follow-ups. 

### Statistical analysis

For statistical analyses, numerical variables were presented as median (q1, q3) according to the normality. We performed the chi-square and Fisher's exact tests to examine the associations between categorical variables or a small sample of categorical variables among studied groups. Analysis of variance was used to compare differences in numerical variables followed by normal distribution between studied groups. Meanwhile, the Mann-Whitney U test compared numerical variables not generally distributed between the two groups. The Statistical Analysis System (SAS) software version 9.4 (SAS Inc, Cary, NC) was used for the data analysis. Then, p < 0.05 was considered significant.

## Results

## Characteristics of patients

Of the 15 enrolled patients bitten by Taiwan cobra, 11 confirmed the species by an atlas of snakes; one patient had taken a picture of the animal, and three by ELISA method. All patients had a final ELISA analysis to confirm the snake species. From the envenomed patients, males accounted for most of this study population (n = 11, 73%), with a median age of 46 (IQR, 39-60) years old. After the snakebite event, ten patients were admitted to EDs in two hours (range: 0.5-2 h), and five were referred from other local hospitals. There was no difference in patients' presentation time (minutes) between the limb swelling group and the wound necrotic group patients (47; IQR 21.5-176.5 vs 55.5; IQR 50-58, p = 0.75). Most of the patients were bitten on the fingers/toes. Almost half of the patients (n = 7) had mild degrees of clinical severity of maximum limb swelling. There were three patients with moderate swelling (two swelling up to the ankle and one swelling up to half of the forearm). Five patients were swelling up to the lower leg (n = 2), knee (n = 1), elbow (n = 1), and upper arm (n = 1), respectively. Eight patients had limb swelling only, and seven patients had tissue necrosis. All patients received antivenom treatment with a medium dose of two vials (IQR 1,4, range 1-10). The wound necrosis group received more antivenom doses than the limb swelling group (4; IQR:2-6 vs 1; IQR:1-2, p = 0.05). Six in eight patients had limb swelling improving in 12 hours in the limb swelling group, and only two in seven patients had their limb swelling begin to be released in 12 hours in the wound necrotic group (one-tailed p = 0.04, Fisher's exact test). 

### Clinical characteristics of cases within limb swelling group and wound necrosis group

There were eight patients in the limb swelling group (Table 2). The degrees of clinical severity of maximum limb swelling were mild (n = 5) to moderate (n = 1) or severe (n = 2). 

**Table 2.** Clinical characteristics of snakebite cases with limb swelling only.

	Limb swelling without wound necrosis							
	Case 1	Case 2	Case 3	Case 4	Case 5	Case 6	Case 7	Case 8
Age/gender	76/M	57/M	28/M	39/M	55/M	45/F	48/M	45/M
Referred from other hospitals	No	No	No	No	No	Yes	Yes	No
Bitten area	Toe	Foot	Toe	Finger	Finger	Toe	Finger	Finger
Severity of max. limb swelling	Foot	Ankle	Knee	Finger	Finger	Toe	Upper arm	Wrist
Clinical severity	Mild	Moderate	Severe	Mild	Mild	Mild	Severe	Mild
Serum venom levels before antivenom (µg/mL)	166.5	21.8	33.1	31	12.4	2.5	-	34.6
Serum venom levels after antivenom (µg /mL)	0.6	0.0	0.0	-	-	0.0	99.9	0.1
Antivenom dose (vial)	1	1	1	1	4	1	2	4
Swelling starts to subside*	Yes	No	No	Yes	Yes	Yes	Yes	Yes
Days in hospital	4	7	4	1	1	1	7	1

*Swelling starts to subside 12 hours after antivenom treatment.

There were seven patients in the wound necrosis group. The degrees of clinical severity of maximum limb swelling were mild (n = 2) to moderate (n = 2) or severe (n = 3) (Table 3). Necrotic lesions were observed in all the wound necrosis group patients when they presented themselves to ED. In most patients, swelling subsided after 12 hours and variations of serum venom concentrations after antivenom treatment are smaller.

**Table 3.** Clinical characteristics of the wound necrosis group.

	Late debridement				Early debridement* in ED		No debridement
	Case 9	Case 10	Case 11†	Case 12	Case 13	Case 14	Case 15
Age/gender	66/M	68/M	28/M	39/M	60/F	39/M	46/F
Referred from other hospitals	No	No	Yes	Yes	No	No	Yes
Bitten area	Toe	Toe	Hand	Buttock	Ankle	Finger	Toe
Severity of max. limb swelling	Lower leg	Foot	Upper arm	Buttock	Lower leg	Forearm	Lower leg
Clinical severity	Severe	Mild	Severe	Mild	Moderate	Moderate	Severe
Initial necrosis**	(+)	(+)	(+)	(+)	(+)	(+)	(+)
Antivenom dose (vial)	4	2	10	6	2	1	6
Time of operation (after bite)	17 hours	3 days	6 days	11.5 hours	1.5 hours	3.5 hours	-
Operation methods	Incision and debridement Wet dressing		Fasciotomy	Incision and debridement	Incision and debridement Wet dressing (case 14)		-
Serum venom levels before antivenom (µg/mL)	64.7	89.3	894.9	-	102.0	342.5	-
Serum venom levels after antivenom (µg/mL)	64.6	78.4	170.2	0.0	75.2	312.7	1.7
Swelling subsides***	No	Yes	No	No	No	Yes	No
Days in hospital	8	3	26	7	7	3	15

*Operation or debridement performed within six hours after snakebites. **Necrotic lesions were observed when they were presented to ED. ***Limb swelling starts to subside 12 hours after antivenom treatment. **†**Received fasciotomy.

The median serum venom concentration was 166.53 (IQR, 64.67-428.75) ng/mL, and there was no statistically difference in pre-antivenom serum concentrations between the limb swelling group and the wound necrosis group (102; IQR:89.3-342.5 vs 192.58; IQR: 33.11-428.75 ng/mL, p = 1.0). After using antivenom, most patients had their serum venom concentrations almost undetectable in the limb swelling group. However, in the wound necrosis group (except for one patient, case 12), the serum venom concentrations after antivenom administration revealed little change compared to the the concentrations before antivenom use. Therefore, the changes in serum venom levels after antivenom were quite different between the limb swelling group and the wound necrosis group (p = 0.0079, Fisher's exact test, Table 4). 

**Table 4.** Comparison of the changes in serum venom concentrations after use of antivenom and antivenom dosage between limb swelling group and wound necrosis group.

	Serum venom concentrations after antivenom*		*p*	Antivenom dose Median (Q1-Q3)	*p*
	Almost reduced to undetectable	Little change			
Limb swelling group	5	0	0.0079**	1 (1-2)	0.05
Wound necrosis group	0	5		4 (2-6)	

*Only patients with records of serum venom concentrations before and after antivenom treatment were included. **Fisher’s exact test.

On the other hand, venom can be detected in the excised tissues, blisters, and wound dressings gauzes. Table 5 demonstrated the concentrations of the whole venom in serum, excised tissues, or wound discharge fluids. In the wound necrosis group, venom concentration in gauzes, blisters, and excised tissues was higher than serum venom levels before the antivenom treatment. In case 10, the venom concentration in gauzes was 43-fold higher than the serum venom levels before antivenom treatment. 

**Table 5.** The concentration of whole venom in serum, excised tissue, and wound discharge fluid.

	Limb swelling without necrosis			Limb swelling with wound necrosis			
	Case 4	Case 5	Case 9	Case 10	Case 11	Case 12	Case 14
Age/gender	39/M	55/M	66/M	68/M	28/M	39/M	39/M
Serum venom levels before antivenom (µg/mL)	218.6	959.8	64.7	89.3	894.9	-	342.5
Samples	Wound	Wound	Gauze	Gauze	Blisters 1-3	Tissue	Gauze
Venom level (µg/mL)	510.1	244.8	976.0	3909.0	1683.49/1890.4/3836.7	559.4	357.3

### Evolution of care in wound necrosis patients

In the past, blebs, blisters, and necrotic tissue may require surgical debridement after several days of snakebites [14]. However, as we observed in case 9, wound necrosis appeared early after snakebites and could easily be extended to other sites of the affected limbs as time went on. Unlike the limb swelling patients, even with antivenom administration, there was still circulating venom in the bloodstream. Therefore, if we can control the snakebite site earlier by debridement and wound wet dressing, less wound necrosis extended to other areas of the affected limbs might be achieved. Based on the above observation, we start early debridement when the patient has cobra envenomation with red to violaceous reticular patches with some central necrotic skin, as demonstrated in case 14. The following section demonstrated three cobra bites related to wound necrosis patients with different wound management methods. 

### No debridement case (case 15)

**Figure 1.**
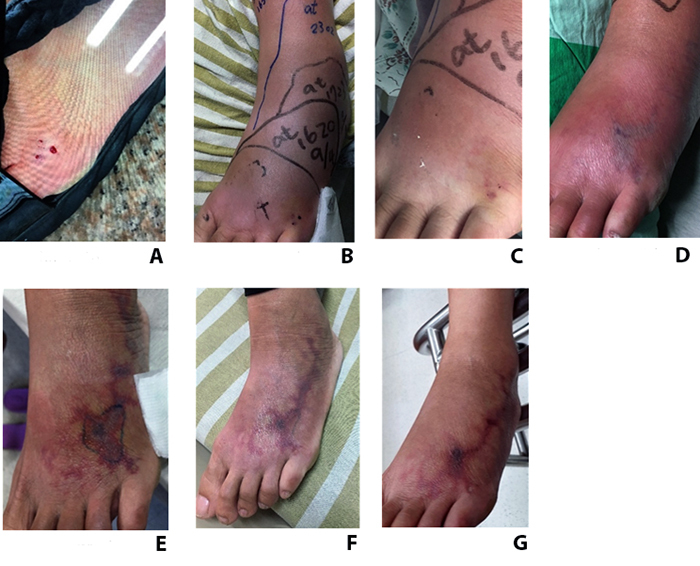
The evolution of a wound that did not undergo debridement. **(A)** A 46-year-old woman was bitten by a cobra on the left 5^th^ toe, presenting pain and foot swelling. **(B, C)** After six hours, red to violaceous reticular patches with some central necrotic tissue were present. **(D, E)** Progressive limb swelling with tissue necrosis on day 7 and day 10. **(F, G)** Wound condition on day 17 and day 30.

The 46-year-old female who refused the suggestion of wound debridement presented to our ED due to a cobra snakebite on the left 5th toe at home (Figure 1A). Limb swelling progressed to the whole left foot 2 hours later even if antivenom was used. In our ED, 4.5 hours after the snakebite, the swelling progressed from the left foot's lateral side to the left leg's lateral lower side with subcutaneous ecchymosis (Figure 1B). The bite site showed red to violaceous reticular patches with central necrotic skin (Figure 1C). The area of the tissue necrosis extended from the bite site to the lateral side of the left ankle with progressive painful sensation even though there was minor limb swelling (Figure 1D, 1E). Even one month after the snakebite, we still could observe the persistently painful necrotic skin over the affected foot (Figure 1F). 

### Late debridement case (case 9)

**Figure 2.**
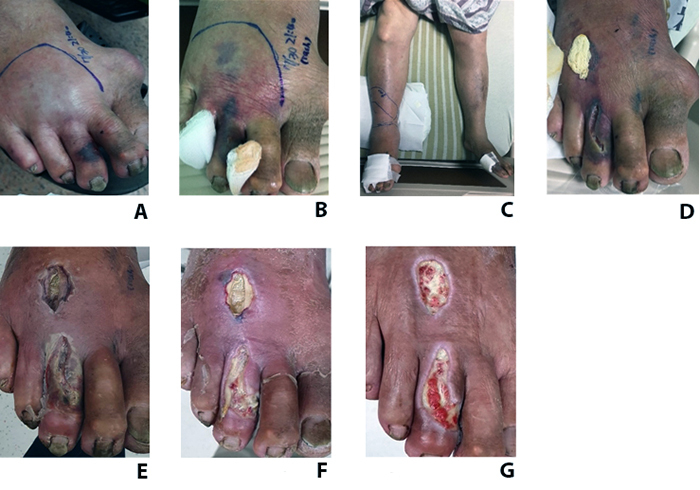
The evolution of wounds in a late debridement case. **(A)** A 66-year-old man was bitten by a cobra on the right 2^nd^ and 3^rd^ toes with necrotic signs within one hour of snakebite. **(B)** The necrotic lesion was observed on the right foot 17 hours later. **(C)** Limb swelling reached the right leg three days later. **(D)** Wound incision and minimal debridement. **(E, F)** Wound condition in one week, one month and two months.

The 66-year-old male presented to our ED due to a snakebite one hour ago. On presentation, there were fang marks over the right 2nd and 3rd toes with central skin necrosis (Figure 2A). There was only mild swelling over the distal portion of the left foot. However, 17 hours after the snakebite, the right toe necrotic skin extended to the right foot (Figure 2B); 3 days later, the swelling extended to the right leg near the knee (Figure 2C). Wound incision and minimal debridement were performed to avoid the tendons being exposed due to less soft tissue on the dorsal foot. After debridement, wound care with normal saline wet dressing was performed, and the area of the necrotic skin was limited in the debridement site (Figure 2D). The wound culture showed *Staphylococcus, Morganella morganii, Bacteroides fragilis,* and *Enterococcus faecalis*. The patient was discharged on day 8 with wound care and an oral antibiotic. Two months later, the debridement wound was healing with much granulation tissue (Figure 2G). 

### Early debridement case (case 14)

**Figure 3.**
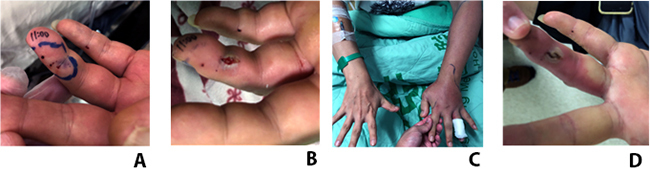
The evolution of wounds in an early debridement case. **(A)** This 40-year-old man presented to our emergency department due to snakebite on the left 4^th^ finger one hour before. **(B)** Wound incision and debridement at ED two hours after the snakebite. **(C)** Swelling on the left forearm on day 2. **(D)** Good wound condition on the 4^th^ day after the bite.

The 40-year-old male came to our ED after being bitten by a cobra snakebite on his left 4th finger. The initial limb swelling was on the finger and reticular patches were noticed surrounding the bite site 1 hour later (Figure 3A). Wound incision and minimal debridement with normal saline wet dressing were performed at ED 2 hours after the bite (Figure 3B). The swelling stopped in the left distal forearm (Figure 3C) and subsided during hospitalization days without adding any dose of antivenom. There was no more wound necrosis skin extension, too. He was discharged on day 4 with good wound condition (Figure 3D). 

## Discussion

In this study, we demonstrated that in the limb swelling group, FN antivenom can decrease circulating venom in five of eight patients (two patients refused to take the blood sample after antivenom treatment). Thus, the FN antivenom could decrease the circulating venom after six hours of antivenom administration in Taiwan cobra envenomed patients presenting limb swelling. Furthermore, clinically, FN antivenom can effectively reverse limb swelling, too. However, in patients with wound necrosis, the FN antivenom cannot eliminate the circulating venom even with a higher antivenom dose than in the limb swelling group. In wound necrosis patients, venom concentration in gauzes, blisters, and excised tissue was much higher than their pre-antivenom serum venom concentrations. This finding was similar to ours and the previous report [15, 16]. In this study, we also revealed that early debridement (within six hours of ED presentation) of the necrotic lesion of Taiwan cobra snakebite patients helps reduce clinical severity and in patients’ wound recovery. 

### The FN antivenom can neutralize serum venom concentration in Taiwan cobra envenomed patients

Antivenom is the only antidote available for the treatment of snakebite patients. According to WHO's guidelines [12], antivenom treatment is indicated in patients who systemic envenoming such as neurotoxicity (bilateral ptosis, external ophthalmoplegia, paralysis, etc.) or local envenoming with local swelling involving more than half bitten limb (in the absence of tourniquet) within 48 hours of the bite; swelling after bites on digits, rapid extension of swelling beyond wrist/ankle within few hours of bites on hand/foot and enlarged tender lymph node draining bitten limb. The TPCC snakebite treatment guideline recommends that FN antivenom is the only therapeutic option for cobra envenomation. Whether in vivo or in vitro, the FH antivenom effectively neutralized neurotoxins of Taiwan cobra venom and can be used to treat the systemic envenomation syndromes caused by Taiwan cobra in patients and animals [9, 15]. Our study had a similar observation in the cobra bite limb swelling patients whose serum venoms were almost neutralized. Therefore, the serum post-antivenom venom levels were diminished after patients were administered various FN antivenom doses, as the previous study reported [8]. However, the above phenomenon was not observed in the wound necrosis group, as demonstrated in this study. There were no differences in pre-antivenom serum venom concentrations between the limb swelling group and the wound necrosis group; however, the wound necrosis group patients had the post-antivenom serum venom levels remained high even though patients were given more antivenom doses than the limb swelling patient group. 

### Wound necrotic site as a venom depot

 Why did the post-antivenom serum venom concentration remain high even though patients were treated with higher antivenom doses? One explanation is that the administrated dose of antivenom is not adequate. However, as demonstrated in the study's first part, the FN antivenom can neutralize the circulating venoms six hours after administering antivenom in Taiwan cobra envenomated limbs swelling patients. In addition, the wound necrosis patient group received more antivenom than the limb swelling patients. Therefore, we need to look for other reasons to explain the minor changes in post-antivenom serum venom concentrations in wound necrosis patients. One possible explanation is that the necrotic wound serves as a venom depot. The venom in the necrotic tissue cannot be neutralized by antivenom since there is poor circulation in the necrotic tissues. Subsequently, the necrotic tissue release venoms into circulation. Therefore, the post-antivenom serum venom concentrations changed only a little when patients were treated with antivenom. Another piece of evidence supporting why we think the necrotic wound tissue is a venom depot is that there is high venom concentration detected in the wound wet dressing gauzes and the excised tissues. Unfortunately, we did not have the serial serum venom and antivenom kinetics data to prove the above hypothesis.

However, by searching the reports in the literature, we can find cases to support our thesis of wound necrotic sites as a venom depot. Lin et al. [15] reported a 68-year-old female suffering from a severe Taiwan cobra envenomation. They described the evolution of the whole venom and cytotoxin A3 concentrations in the patient's local wound discharge (bulla). The authors found both whole venom and cytotoxin A3 concentrations in the patient's bulla were reduced on day three after six vials of antivenom treatment. However, on day 4, both whole venom and cytotoxin A3 concentrations in the bulla rebounded, and more antivenom was given. On day 5, whole venom and cytotoxin A3 concentrations in patients’ bulla were very low until the patient received the wound debridement procedure. Therefore, this study demonstrated that patients' bullae served as a venom depot and local debridement could reduce the venom load.

In our study, case 14 received wound debridement and only one vial of antivenom soon after ED arrival. His limb swelling progressed to the forearm on the 2^nd^ day after the snakebite. However, even with no more antivenom added to treat his limb swelling, the swelling wholly resolved with wound care with normal saline wet dressing only. We believed that the normal saline wet dressing method could remove venom from the patient wound; thus, the limb swelling resolved. Venoms presented in the wound wet dressing gauzes provided evidence of a wound necrotic site as a venom depot.

On the contrary, patients with late debridement and no debridement cases of the wound necrosis group need more antivenom doses than one vial of antivenom to resolve their limb swelling. Based on the above observation, we believe that the thesis of a wound necrotic site as a venom depot is highly possible. We should remove the necrosis tissue or aspirate the bulla fluid to reduce the venom load to prevent further envenomation and better wound healing.

### The persistence of Taiwan cobra venom in local tissue and the need of early wound debridement

As we demonstrated in this study, in our previous and other studies, the rate of wound necrosis after cobra bites was high, and up to 30%- 70% of patients needed to receive surgical interventions despite high antivenom dosage [2, 7, 9, 17]. According to the wound management section of the snakebite section of WHO's guidelines for the management of snakebites [13], it was suggested that “once frank skin necrosis (demarcated, hypo/hyperpigmented areas with an odor of putrefaction) is detected, surgical debridement is indicated to remove the risk of anaerobic sepsis.” It was also suggested that “Necrotic tissue demands early surgical debridement and split-skin grafting” in treating the bitten part of the guideline. However, the timing of surgical procedures seems late in the clinical setting. It had been reported that the first operation procedure was performed at a median of 3.5 days (IQR, 2-6 days) [7]. However, the timing of the debridement was at least partially determined by the appearance of demarcation between necrotic and healthy tissues and maybe thus delayed. The presence of tissue necrosis may be later than our studies and therefore limits the early application of wound debridement in previous studies.

Our study revealed that the FN antivenom could neutralize the circulating venoms in Taiwan cobra envenomated limb swelling patients. However, even after administering a large dose of antivenom, various venom concentrations were detected in gauzes, blisters, and excised tissues in patients with wound necrosis. Cytotoxins of the three-finger toxin family are thought to cause wound necrosis after cobra bites [18, 19, 20]. The cytotoxin could conjugate with the tissues in the biting site and has thus formed a high tissue affinity, making it resist being neutralized by antivenom [21]. This is quite consistent with our case 15, the no debridement case. One month after the snakebite, the wound necrosis of her affected foot is still well observed even after a large amount of antivenom is administrated. Thus, the high tissue affinity of cytotoxin of Taiwan cobra venom could lead to the natural limitation of antivenom effects in treating snakebite patients.

Furthermore, the locally retained venom toxins could cause more severe local tissue damage beyond the biting site. Thus, based on the observation of the persistent existence of Taiwan cobra venom in local tissue and wound exudate, blisters as our studies observed, we suggested early wound debridement, aspirating the venom-containing blister fluids, or wound care with normal saline gauzes as measurements of decontaminating the venom might be valuable in reducing local tissue and having better wound healing in Taiwan cobra envenomation patients. Similarly, Zeng et al. [22] reported that multiple small incisions combined with negative pressure wound therapy proved effective for controlling the release of inflammatory cytokines, reduction in limb swelling, and complication rates in *Protobothrops mucrosquamatus* bite envenomation patients.

Another reason to perform an early debridement in Taiwan cobra envenomated patients is to reduce the possibility of progression of wound necrosis caused by a wound infection. In Taiwan, cobra bites caused more severe bacterial infections than other snakebites [23], as our late debridement case demonstrated. The infection rate of Taiwan cobra bites has varied from 20% [23, 24] to as high as 70% [25, 26]. Early radical surgical debridement and empirical broad-spectrum antimicrobial treatment remain the cornerstones of therapy in necrotizing soft-tissue infections [27]. Therefore, early wound debridement might minimize the wound with more tissue being preserved, prevent disability, and reduce the length of hospitalization [22, 28].

### The decision-making process of local debridement and excision, and choice of antibiotics in Taiwan cobra bite patients

The decision-making of different debridement procedures and excision depends on patients' presentation time after snakebites to ED and snakebite site. In our study, most patients had distal limb snakebite (toe, foot, finger, and hand); less soft tissue and easy tendon or vascular exposure are the major problems on these snakebite sites. In early ER admission with distal limb snakebite patients, less soft tissue excision and incision with wet dressing can reduce tissue loss and avoid tendon or vascular exposure (Figure 3). However, in suppose delayed ED admission with a distal limb snakebite patient, the necrotic tissue may extend (as in our case 9). In that case, limited wound excision should be done, and a normal saline wet dressing can also achieve a good result (Figure 2). If the snakebite site is above the hand or foot (like arm, leg, or buttock), wide excision and debridement are suitable and thus can make less snake envenomation extension and good wound healing.

The choice of empirical antibiotics should be according to the previous results of wound bacterial cultures. The most found pathogens were aerobic gram-negative bacteria *M. morganii* and gram-positive bacteria *Enterococcus* spp. In patients who underwent several surgical procedures, anaerobic bacteria such as*Bacteroides fragilis*,*Providencia rettgeri*,*Proteus vulgaris*, and*Serratia marcescens*were observed [24]. Therefore, appropriate antibiotics for first-line monotherapies are gentamicin, ceftriaxone, ciprofloxacin, or levofloxacin. If patients received multiple surgical procedures, anaerobic wound infection would be encountered; metronidazole, augmentin, and piperacillin/tazobactam are recommended. However, ureidopenicillin, such as piperacillin, should be reserved for patients at risk of*Pseudomonas*spp. infection.

### Doses required to treat Taiwan cobra envenomation patients

In clinical practice, the doses of antivenom used to treat Taiwan cobra envenomation were highly variable. A wide range of dosages applied to treat Taiwan cobra envenomated patients in the literature was evident, too [7, 8, 9]. Such variation in dosage may be primarily explained by the different amount of venom injected at each snakebite episode or a rebound in serum venom levels after initial antivenom therapy in wound necrosis patients. In this study, we observed two different situations of the FN antivenom in treating Taiwan cobra envenomated patients. First, the FN antivenom can neutralize most circulating venom six hours after antivenom administration in Taiwan cobra envenomated limb swelling patients. Second, the FN antivenom cannot eliminate circulating venom in patients with wound necrosis, even with a high antivenom dose. Therefore, for patients with wound necrosis whose limb swelling did not subside ideally, physicians would add more doses of antivenom. Thus, a high variation of antivenom dosage could have resulted. Venom in blisters and excised tissues was evident in the wound necrosis patients. As we mentioned above, venom in excised tissues is due to the high tissue affinity of Taiwan cobra venom. It thus could lead to natural limitation of antivenom effects in treating wound necrosis patients. Therefore, we need to consider different dosing schedules for these two kinds of patients. 

Lacking a well-designed clinical severity system to guide the use of antivenom might also be another factor causing the high variation of antivenom dosage. In limb swelling patients, we need to develop a severity grading system or clinically valuable useful biomarkers to provide a more objective instrument for evaluating the severity and progression of snakebites envenomation to guide the use of antivenom dosage. 

Regarding the wound necrosis patients, as we mentioned above, we thought early debridement and wound wet dressing management should be arranged early (maybe within six hours after the patient presentation in ED). We recommend using at least one vial of antivenom or more to combat the circulating venom, conquer the venom-induced systemic effects and administer more antivenom if deteriorating neurotoxic or cardiovascular signs (after one hour) [13] and rapid progressive limb swelling (after six hours) of the first antivenom dose. The TPCC guideline stated only a rough range of dosage of FN antivenom for Taiwan cobra bites patients and no mention of any recommendation in treating Taiwan cobra bites-related wound necrosis. In this study, we offered our treatment protocol for limb swelling-only patients and proposed early debridement of the necrotic wound. However, we need more studies to achieve the precision use of antivenom instead of administrating antivenom on physicians' free evaluation of the evidence to avoid wasting valuable and scarce antivenom stocks.

### Possible clinical applications of our study findings in other *Naja* spp. bites

Envenomation caused by different *Naja* species such as *Naja siamensis* (Thai spitting cobra) [29],*Naja kaouthia*(monoacetate cobra) [30],*Naja naja*(Indian cobra) [31], and*Naja mossambica*(Mozambique spitting cobra) [32] share the common features of local tissue swelling, inflammation, infection, and significant tissue necrosis as does Taiwan cobra bites. Although there are different venom compositions among these *Naja* species; however, the venom toxins that induced tissue necrosis are quite similar and can be strongly recognized by the FN antivenom [33]. Rha et al. [34] compared various non-cobra snakebites patients who had received debridement without antivenom administration due to a positive skin reaction test and patients who received antivenom and delayed debridement and found that there was less wound infection resulting in the early debridement patients. Thus, we believe the findings and suggestions of this study may have substantial potential for improving treatment strategies in antivenom use and wound management in snakebite patients. 

### Limitations

The small sample size was the major limitation. Therefore, this study should be presumed as a preliminary report to document the changes in serum venom concentrations before and after the administration of antivenom, the existence of venom in necrotic tissues, blisters, and wound wet dressing gauzes, and the possible role of early wound debridement in Taiwan cobra bite patients. A well-designed clinical trial is needed to clarify our proposed treatment suggestions. 

## Conclusions

 Based on the limited data we provide here, the FN antivenom can efficiently neutralize the circulating cobra venom in limb swelling patients without wound necrosis and antivenom effectively reverse limb swelling. We proposed that the necrotic wound site may release venom into circulation and early wound debridement could improve therapeutic outcomes. Further research is required to prove this proposal before its full implementation in patient care.
